# Editorial: Immune system disorders: from molecular mechanisms to clinical implications

**DOI:** 10.3389/fimmu.2024.1498830

**Published:** 2024-10-08

**Authors:** Marisa M. Fernandez, Ruben D. Motrich, Matías Ostrowski, Mauricio De Marzi

**Affiliations:** ^1^ Universidad de Buenos Aires, Facultad de Farmacia y Bioquímica, Departamento de Microbiología, Inmunología, Biotecnología y Genética, Cátedra de Inmunología, Buenos Aires, Argentina; ^2^ Instituto de Estudios de Inmunidad Humoral, Universidad de Buenos Aires-Consejo Nacional de Investigaciones Científicas y Técnicas (CONICET), Buenos Aires, Argentina; ^3^ Federation of Clinical Immunology Societies (FOCIS) Center of Excellence, Centro de Inmunología Clínica de Córdoba (CICC), Córdoba, Argentina; ^4^ Centro de Investigaciones en Bioquimica Clinica e Inmunologia (CIBICI)-Consejo Nacional de Investigaciones Científicas y Técnicas (CONICET), Facultad de Ciencias Químicas, Universidad Nacional de Córdoba, Córdoba, Argentina; ^5^ Instituto de Investigaciones Biomedicas en Retrovirus y Síndrome de Inmunodeficiencia Adquirida (SIDA), Facultad de Medicina, Universidad de Buenos Aires/CONICET, Buenos Aires, Argentina; ^6^ Grupo de Investigaciones Básicas y Aplicadas en Inmunología y Bioactivos (GIBAIB), Instituto de Ecología y Desarrollo Sustentable (INEDES), (Universidad Nacional de Luján – CONICET), Luján, Buenos Aires, Argentina; ^7^ Universidad Nacional de Luján, Departamento de Ciencias Básicas, Luján, Buenos Aires, Argentina

**Keywords:** immune system disorders, dysfunction, autoimmune diseases, autoinflammatory diseases, immunodeficiency, immunosuppression, immune therapy, immune modulation

**Figure 1:**
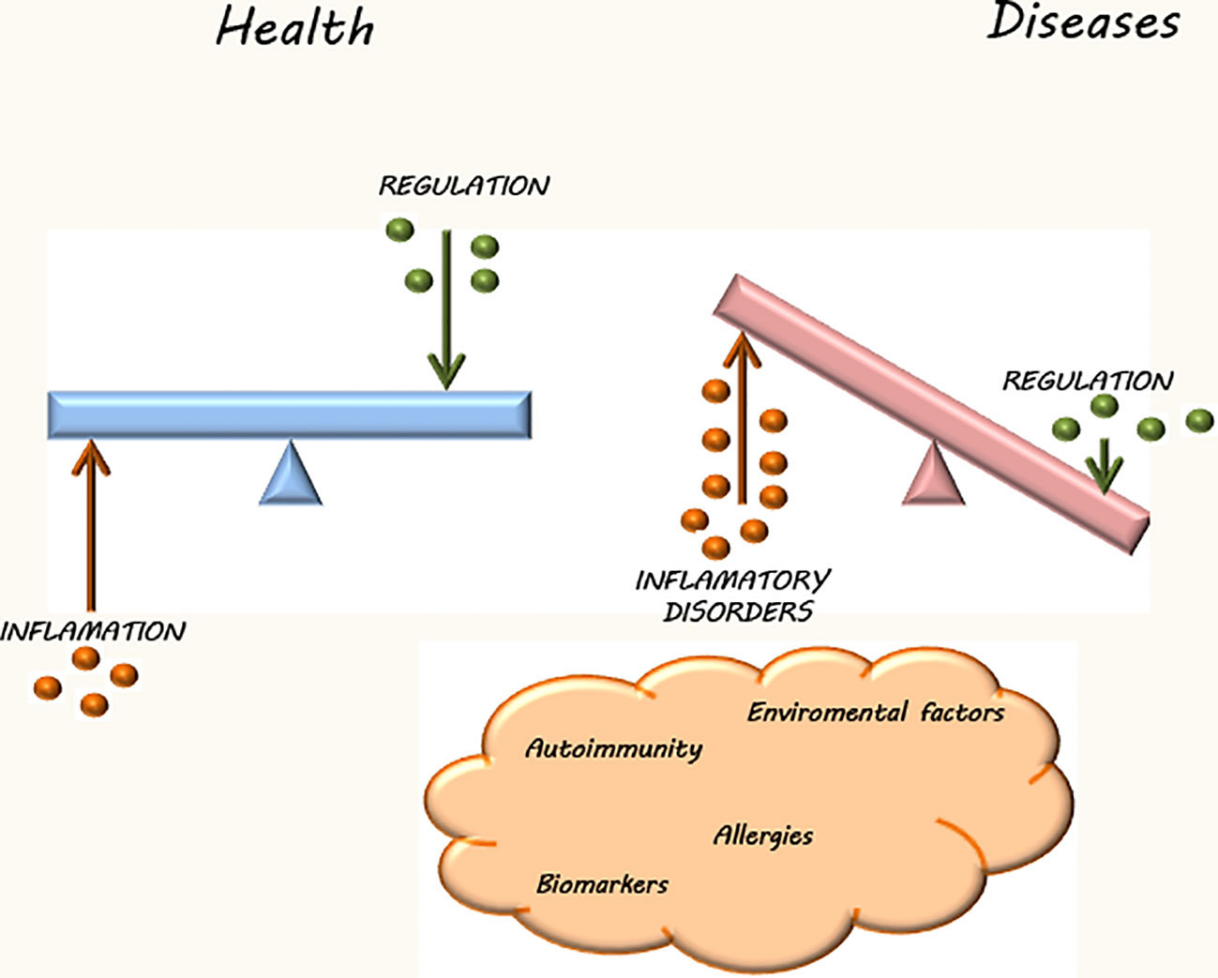
The immune system has a fundamental role in immune surveillance to prevent the development of tumor cells and inflammatory alterations or in immunological tolerance processes. The pathological consequences of its deregulation include inflammatory, autoimmune and immunosuppressive diseases, among others.

In recent years, our understanding of the immune system has expanded significantly, highlighting its crucial role not only in the defense against pathogens, toxins and tumor cells but also in controlling inflammation and inducing tolerance to self- and non-self-antigens (1, 2). Reported evidence during the last decades has allowed a better understanding of the pathogenic consequences of immune system dysfunction, including the development of immunodeficiencies, inflammatory and autoimmune disorders, among others (3). Moreover, current knowledge about immunoregulation has paved the way to better prevent or treat severe inflammatory conditions such as transplant rejection and hypersensitivities. However, mechanisms underlying immune deregulation are highly variable depending on the pathology (systemic chronic inflammatory diseases, autoinflammatory diseases, autoimmune disorders, immunodeficiencies, hypersensitivities) and the host. Given the diverse range of disorders resulting from excessive, inefficient, or inadequate immune responses, the study of factors involved in immune system deregulation has garnered significant attention in recent decades (**Figure 1**). In this Research Topic, some review articles has focused on different autoimmune diseases (AIDs) and treatments. Cavalcante et al. conducted a literature review on emerging biological drugs for treating Myasthenia gravis and emphasized the crucial importance of precision medicine for these patients, given the variability in the efficacy of current therapies. Moreover, Zinellu et al. performed a systematic review about the role of circulating concentrations of bilirubin as a biomarker of rheumatic diseases (RDs) revealing that patients show significantly lower concentrations of total bilirubin, conjugated bilirubin, as well as of the active antioxidant and anti-inflammatory unconjugated bilirubin. Consequently, they propose them as potential biomarkers of antioxidant and anti-inflammatory capacity in patients bearing rheumatoid arthritis (RA), systemic lupus erythematosus (SLE), primary Sjögren syndrome, and myositis. Besides, Chen et al., described the composition, biological function, and regulation of N 6 -methyladenosine (m 6 A) in the immune microenvironment and its role in various immune diseases such as inflammatory enteritis and SLE, providing new targets and directions for their treatment. A mini review presented by Lazarevic et al. describes that complete Freund’s adjuvant (CFA) used for the induction of experimental autoimmune encephalomyelitis (EAE) (a model of multiple sclerosis –MS-) is a confounding factor due to the multiple effects it causes in animals. For this reason, EAE variants without CFA are highlighted as valuable tools to study the pathogenesis of MS.

Another review by trigger Zhao et al. highlights how significant changes in inflammatory factors can drive phenotypic transformations of the disease, shifting from autoimmune hematopoietic insufficiency (AHF) to myeloid neoplasms (MN). This suggests that the autoimmune responses observed in AHF may act as an antileukemic mechanism, and their suppression could potentially trigger the development of MN. In addition, the effectiveness of IL-1 pathway inhibitors for treating PSTPIP1-associated inflammatory diseases (PAID) was examined in a systematic review by Sanz-Cabanillas et al. While anakinra and canakinumab have demonstrated potential in managing conditions such as sterile pyogenic arthritis, pyoderma gangrenosum and acne (PAPA), and PSTPIP1-associated myeloid proteinemia inflammatory (PAMI) syndromes, there is limited information available regarding the adverse effects of these treatments. As anticipated, regulatory T cells (Tregs) feature prominently in this Research Topic. Ou et al. provide a comprehensive summary of Tregs’ modulatory effects, emphasizing their critical interactions with innate immune effectors and their influence on disease development. They also discuss the potential therapeutic applications of Tregs in the context of autoimmunity, allogeneic transplants, and various common medical conditions where organ and tissue damage is primarily driven by inflammatory processes.

This Research Topic also includes several original research articles that expand our current knowledge on various aspects of several different immune-mediated diseases, such as allergic rhinitis, rheumatoid arthritis, cardiovascular disease, autoimmune thyroid disease, idiopathic pulmonary fibrosis, ulcerative colitis, psoriasis, Sjögren’s syndrome, chronic inflammatory demyelinating polyradiculoneuropathy, systemic lupus erythematosus, and ankylosing spondylitis, among others.

Chen et al. performed a study utilizing Mendelian randomization (MR) to explore the relationship between immune cell phenotypes and metabolite levels in allergic rhinitis (AR). Notably, three immune cell phenotypes were identified as protective factors for AR: naïve CD8br %CD8br cells, CD3 in CD39+ activated Tregs, and HVEM in CD45RA-CD4+ cells. Additionally, authors identified three metabolites as risk biomarkers for AR: N-methylhydroxyproline, N-acetylneuraminate, and 1stearoyl-2-arachidonoyl-gpc (Chen et al.).

Different attention-grabbing reports focused on rheumatoid arthritis (RA), a common and debilitating chronic systemic autoimmune disease. Given that heart failure (HF) has emerged as the second leading cause of cardiovascular death among RA patients, Kadier et al. investigated the association between HF and RA. While their cross-sectional study of the U.S. population revealed a significant link between the two, similar findings were not observed in the European population through Mendelian randomization analysis, suggesting that further research is needed on this topic. Additionally, Chang et al. utilized machine learning to analyze RA subtypes, identifying three distinct categories based on upregulated differentially expressed genes (DEGs): RA subtype A, which is enriched in pathways related to neutrophil activation; RA subtype B, associated with IFN signaling; and RA subtype C, driven by CD8+ T cells. These findings offer valuable insights for patient stratification, potentially paving the way for enhanced molecular diagnostics and therapeutic strategies in the future. It is well established that the neutrophil-to-lymphocyte ratio (NLR) serves as a biomarker for systemic inflammation and immune activation. In their study, Zhou et al. demonstrate that RA patients with a higher NLR face an increased risk of all-cause and cardiovascular mortality compared to those with a lower NLR. They suggest that NLR could be an inexpensive and readily accessible prognostic marker for RA. Disruption of bone metabolism is another important factor altered in RA. Adami et al. conducted a cross-sectional case-control study to explore associations between biomarker levels and clinical variables. The authors identified a distinct bone profile in RA, characterized by changes in bone density and unique bone biomarker patterns, which may provide valuable insights for enhancing the management of bone involvement in RA. Noteworthy, a controversial association between hypothyroidism and RA has been proposed. Interestingly, Peng et al. report a potential causal association of hypothyroidism with RA. Analyzing gene expression data in several tissues, authors observed a genetic associations between hypothyroidism and RA, particularly in local genomic regions. They identified TYK2, IL2RA, and IRF5 as shared risk genes for both hypothyroidism and RA.

Evidence suggests a potential relationship between Parkinson’s disease (PD) and various autoimmune diseases (AIDs). Yang et al. conducted a bidirectional Mendelian randomization analysis to explore the causal associations between PD and a range of AIDs. Notably, their findings indicate a potential positive correlation between genetically determined PD and the incidence of type 1 diabetes (Yang et al.). Besides, another MR study revealed the close association between immune cells and generalized anxiety disorder (GAD) through genetic methods, thereby offering direction for future clinical research (Ma et al.). Autoimmune thyroid disease (AITD) is among the most common thyroid disorders. A MR analysis by Yao et al. identified seven causal associations between inflammatory cytokines and AITD. Elevated levels of TNF-β and reduced levels of SCGF-β were associated with an increased risk of Graves disease (GD). Conversely, high levels of IL-12p70, IL-13, and IFN-γ, along with low levels of MCP-1 and TNF-α, indicated a higher risk of Hashimoto thyroiditis (HT) (Yao et al.).

Idiopathic pulmonary fibrosis (IPF) is a progressive lung function deteriorating condition associated with high mortality. By using microarray datasets, Hu and Xu analyzed the relationships between differentially expressed genes (DEGs) and IPF. Authors identified 486 highly expressed genes and 468 lowly expressed genes. However, MR analysis identified six significantly co-expressed genes associated with IPF that participate in essential biological processes and pathways, including macrophage activation and neural system regulation. Authors emphasize the potential of targeting specific molecular pathways for the treatment of IPF, laying the foundation for further research and clinical work (Wenzhong Hu and Yun Xu).

Another study using machine learning algorithms identified 108 differentially expressed mitochondria-related genes (DE-MiRGs) in the colonic mucosa of ulcerative colitis (UC) patients (Zhang et al.). Results revealed a significant enrichment in pathways associated with mitochondrial metabolism and inflammation. Moreover, valuable MiRGs diagnostic models with predictive capabilities and therapeutic implications for UC were developed based on 17 signature genes (Zhang et al.). Similarly, Zhou et al., employed machine learning and analysis of DEGs in the skin of patients with psoriasis and atopic dermatitis. They identified four potential diagnostic genes and validated the results using real-time quantitative polymerase chain reaction (RT-qPCR) and immunohistochemistry. In this way, they propose the CCNE1 gene as the one with the greatest diagnostic value. Psoriasis is a highly heterogeneous autoimmune skin disease that affects approximately 2-3% of the global population and lacks effective treatment options. In their study, Ishimoto et al. identify environmental antigens that may trigger autoimmunity in psoriasis due to TCR polyspecificity, including peptides from wheat, *Saccharomyces cerevisiae*, microbiota, tobacco, and several pathogens. They discovered that the same CD8+ T cells can recognize both autoantigens and environmental antigens, suggesting that a wheat-free diet may alleviate psoriasis symptoms in some patients. Besides, Zhang et al. identified 163 differentially upregulated genes in lesional skin from psoriatic patients using microarray datasets. Interestingly, they described three skin subtypes (A-C subtypes). Subtype A is related to activated immune cells and pathways related to inflammation, subtype B was modestly activated in all the signaling pathways, and subtype C exhibited high levels of stromal cells and signaling pathways activation associated with tissue proliferation. Moreover, Xgboost classifier predicts that subtypes A and B would respond well to methotrexate and interleukin-12/23 inhibitor treatments, while subtype C would have excellent outcomes after tumor necrosis factor-α inhibitors (etanercept) and interleukin-17A receptor inhibitors (brodalumab) therapies (Zhang et al.). The systemic immune-inflammation index (SII), as measured by lymphocyte, neutrophil and platelet counts in peripheral blood, is regarded as a reliable indicator of inflammatory state. By multivariate linear regression analysis, Zhao et al. report a significant positive correlation between psoriasis and elevated SII. Nonetheless, authors are cautious about their findings and suggest the need for further large-scale prospective studies to validate their results (Zhao et al.).

Chronic pelvic pain syndrome is a prevalent urological condition affecting young adult men (4). Patients typically present signs and symptoms of chronic inflammation in the absence of a detectable infectious cause. In this Research Topic, Salazar et al. report evidence indicating that CD8 T cells do not play a major role in the pathogenesis of chronic prostatitis and pelvic pain development using an animal model of Experimental Autoimmune Prostatitis (EAP), which has been largely used to study CPPS pathogenesis (5). Sjögren’s syndrome is another intriguing prevalent autoimmune condition. Park et al. report that patients with higher T cell counts in the infiltrates of lymphocytic foci may face a two-fold increased risk of severe disease flares. Additionally, they found a weak but positive correlation between B cell and CD4+ T cell counts and the EULAR Sjögren’s Syndrome Disease Activity Index (ESSDAI) (Park et al.).

According to the latest guidelines on chronic inflammatory demyelinating polyradiculoneuropathy (CIDP), patients with CIDP with anti-neurofascin 155 (NF155) antibodies are referred to as autoimmune nodopathy (AN), an autoimmune disorder distinct from CIDP. In a very original study, Zhang et al. provide evidence indicating that patients with AN with anti-NF155 antibodies differed from serologically negative patients with CIDP in terms of clinical characteristics such as onset age, limb weakness, sensory disturbance, ataxia, and multiple motor (Zhang et al.).

A retrospective analysis of systemic lupus erythematosus (SLE) was performed by Geng et al. to assess putative correlations with neuropsychiatric systemic lupus erythematosus (NPSLE). Authors found that 82.4% of psychiatric manifestations were attributed to SLE. Remarkably, mood disorders positively correlated with anti-cardiolipin (ACL) and anti-β2 glycoprotein I (-β2GP1) antibodies (Geng et al.). Finally, Zuo and Li carried out a MR analysis to confirm causal relationships between ankylosing spondylitis (AS) and five mental health conditions including major depressive disorder (MDD), anxiety disorder (AXD), schizophrenia (SCZ), bipolar disorder (BIP), and anorexia nervosa (AN). The authors found that ankylosing spondylitis (AS) may be causally linked to an increased risk of developing schizophrenia (SCZ) and anorexia nervosa (AN), offering new insights for risk management and preventive interventions for mental disorders in patients with AS (Zuo and Li).

This Research Topic aims to enhance our understanding of the cellular and molecular mechanisms underlying diseases associated with immune dysregulation. Much of the current focus is on exploring the interactions between immune dysregulation and factors such as age, sex, genetics, nutritional status, past infections, and environmental influences. These insights are expected to pave the way for the development of effective, targeted therapies for these conditions. However, further knowledge is needed regarding the consequences of the various processes that impact the immune system.
